# Effects of Low-Load, High-Repetition Resistance Training on Maximum Muscle Strength and Muscle Damage in Elite Weightlifters: A Preliminary Study

**DOI:** 10.3390/ijms242317079

**Published:** 2023-12-03

**Authors:** Dong-Chul Yeom, Dong-Joo Hwang, Woong-Bae Lee, Joon-Yong Cho, Jung-Hoon Koo

**Affiliations:** 1 Department of Weightlifting, Korea National Sport University, Seoul 05541, Republic of Korea; mangjark@knsu.ac.kr; 2 Exercise Biochemistry Laboratory, Korea National Sport University, Seoul 05541, Republic of Korea; dongzoo87@knsu.ac.kr (D.-J.H.); chojy86@knsu.ac.kr (J.-Y.C.); 3 Sport Science Institute, Korea National Sport University, Seoul 05541, Republic of Korea; 4 Department of Beauty Health Science, Shinhan University, Euijeongbu 11644, Republic of Korea; kffsg@shinhan.ac.kr; 5 Department of Exercise Training for Health Care & Management, Korea National Sport University, Seoul 05541, Republic of Korea

**Keywords:** weightlifting, training load, training repetitions, muscle hypertrophy, muscle damage

## Abstract

This study aimed to assess the impact of different resistance training (RT) loads and repetition on muscle damage, intramuscular anabolic signaling, and maximal muscle strength (MMS) in weightlifters. Eighteen male weightlifters were randomly assigned to 8 weeks of supervised RT regimes: high-load, low-repetition (HL), low-load, high-repetition (LH), and combination of HL and LH (COMBI). All groups exhibited a significant increase in skeletal muscle mass (SMM) and growth hormone levels, which ultimately contributed to improvement in MMS as indicated by 1-repetition maximum in the back squat and back muscle strength. Notably, while there were no significant changes in the mTOR protein, the phosphorylation of phosphorylation of p70 ribosomal protein S6 kinase 1 (p70S6K1), eukaryotic initiation factor 4E-binding protein 1 (4E-BP1), and eukaryotic elongation factor 2 (eEF2), which are involved in muscle cell growth, was significantly affected by the different training regimens. More importantly, LH-RT led to a significant reduction in muscle damage markers, creatine kinase (CK) and lactate dehydrogenase (LDH), suggesting reduced recovery time and fatigue. Our results demonstrated that the LH-RT paradigm could be a viable alternative for weightlifters to enhance MMS and muscle hypertrophy similar to HL-RT, while reducing RT-induced muscle damage, ultimately contributing to the enhancement of exercise performance.

## 1. Introduction

MMS is a critical factor in the determination of high-intensity anaerobic sport performance, such as weightlifting, and therefore is heavily focused upon during training. As a general practice, competitive weightlifters train with relatively high-loads and low-repetitions (HL; ≥80% of 1-RM) during RT sessions, which has been shown to be an effective programming method for increasing maximum muscle strength and power [1]. However, long-term excessive HL-RT coupled with insufficient recovery is known to cause overtraining, which can negatively impact muscle recovery, mood, and hormone levels; potentially leading to augmented muscle damage and results in diminished performance [2,3]. Thus, an RT program that can increase maximum muscle strength while minimizing the negative side effects of HL-RT in weightlifting athletes is needed.

Recent studies suggest that low-load, high-repetition (LH)-RT produce effects comparable to HL-RT, promoting acute muscle protein synthesis and hypertrophy [4,5]. This response may be attributed to LH-RT’s capacity to recruit fast-twitch motor units and stimulate myofibrillar protein synthesis mediated by activating the protein kinase B and mammalian target of rapamycin (AKT/mTOR) signaling pathway [6]. According to the previous findings, the pathway regulates the phosphorylation of downstream targets such as p70S6K1, 4E-BP1, and eEF2, which are crucial for muscle hypertrophy and protein synthesis [6,7,8,9,10]. Thus, the evidence suggests that LH-RT has the potential to induce muscle hypertrophy, thereby enhancing maximum muscle strength while possibly mitigating excessive muscular damage associated with HL-RT.

However, previous studies have not offered sufficient evidence to fully support the implementation of LH-RT in elite athletes, specifically due to the lack of evidence regarding the duration of RT and the variable effects of training status (untrained vs. well-trained). For example, previous studies investigated the effects of an acute bout of LH-RT on the mTOR signaling pathway [11,12], and they provided evidence of pathway activation. The responses to an acute bout of RT varied drastically between the investigations. In addition, it is well accepted that RT-mediated muscle hypertrophy is a long-term phenomenon, and therefore, more appropriate to investigate the effects of chronic RT rather than following an acute stimulus. Furthermore, the previous studies utilized various participant populations ranging from entry-level to novice (elderly and/or untrained) [4,6,9,10,13]. Indeed, the effect of RT-induced changes in muscle strength alter primarily as neuronal adaptation during the early stages of training for untrained subjects [14]. However, for well-trained individuals, the effects manifest progressively and more slowly as experience and training status [5,15]. These results are therefore confounding for elite weightlifters and their coaches who may consider applying LH-RT to improve exercise performance. Thus, it is necessary to confirm whether chronic LH-RT exerts a positive effect on MMS and its related intramuscular anabolic mechanisms in elite weightlifters. However, the precise mechanisms underlying the effects of chronic LH-RT on muscle hypertrophy-related factors and MMS in elite weightlifters remain unclear.

Thus, the purpose of our study was to investigate whether 8 weeks of three types of RT [HL, LH, and a combination of HL and LH (COMBI)] could produce positive effects on body composition, anabolic hormones, and AKT/mTOR signaling in elite weightlifters. Furthermore, our study investigated MMS using 1-RM (back squat and snatch), back muscle strength (BMS), maximal isometric strength of the trunk muscles, and markers of muscle damage such as CK and LDH to verify whether these types of RT affected weightlifting-related performance and RT-induced muscle damage.

## 2. Results

### 2.1. Body Composition and Anabolic Hormones

No significant differences were observed between groups with respect to body composition. However, SMM was significantly increased following training in all groups when compared with pre-RT COMBI (*p* = 0.046), LH (*p* = 0.046), and HL (*p* = 0.027) groups (Table 1). Next, we measured insulin, IGF-1, and GH levels to assess the effects of the RT regimens on anabolic responses. However, there were no significant differences between groups for all the measured variables (Table 1). GH increased significantly post-RT compared with pre-RT in COMBI (*p* = 0.046), LH (*p* = 0.028), and HL (*p* = 0.028) groups.

### 2.2. Maximal Muscle Strength

**Figure 1:**
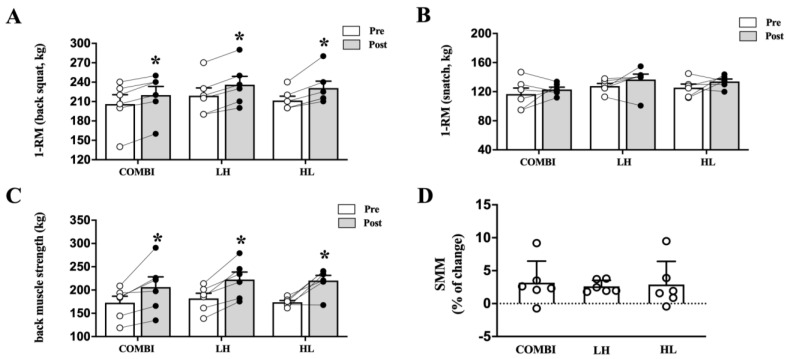
Effect of three RT regimens on maximum muscle strength and percentage change in skeletal muscle mass in elite weightlifters. (**A**) 1−RM of back squat, (**B**) 1−RM of snatch, (**C**) back muscle strength, and (**D**) percentage change in skeletal muscle mass (SMM) following 8 weeks of three RT regimens. Each circle, with white and black colors, represents individual data points before and after RT, respectively. Bars represent mean ± SD (n = 6 per group). * *p* < 0.05 from Pre to Post.

MMS measurements via 1-RM of back squat, snatch, and BMS revealed no significant difference between groups (Figure 1). Back squat and BMS 1-RM were significantly increased post-RT compared with pre-RT in the COMBI, LH, and HL groups (back squat: *p* = 0.026, *p* = 0.026, *p* = 0.026, respectively, and BMS: *p* = 0.026, *p* = 0.026, *p* = 0.026, respectively, Figure 1A,C). Next, we measured the change in SMM from baseline, which was +2.9%, +3.2%, and +2.6% in the COMBI, LH, and HL groups, respectively (Figure 1D).

### 2.3. Phosphorylation of AKT and mTOR Protein

**Figure 2:**
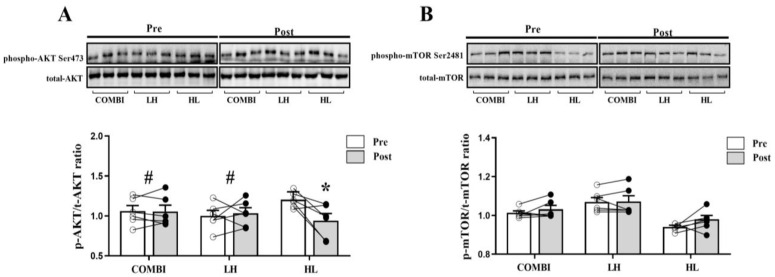
Effect of three RT regimens on phosphorylation of AKT and mTOR in elite weightlifters. (**A**) Representative western blots and quantification of phospho-AKT to total-AKT (p-AKT/t-AKT) ratio and (**B**) phospho-mTOR to total-mTOR (p-mTOR/t-mTOR) ratio. Each circle, with white and black colors, represents individual data points before and after RT, respectively. Bars represent mean ± SD (n = 6 per group). * *p* < 0.05, from Pre to Post. # *p* < 0.05, compared with HL group.

As shown in Figure 2A, we identified significant differences in the p-AKT/t-AKT ratio between groups (*p* = 0.036). The p-AKT/t-AKT ratio was significantly lower in the HL group compared to the COMBI and LH groups (*p* = 0.049, *p* = 0.036, respectively), and it was significantly decreased post-RT compared to pre-RT only in the HL group (*p* = 0.046). Unexpectedly, there were no significant differences in the p-mTOR/t-mTOR ratio between groups over time (Figure 2B).

### 2.4. Phosphorylation of p70S6K1, 4EBP1, and eEF2 Protein

**Figure 3:**
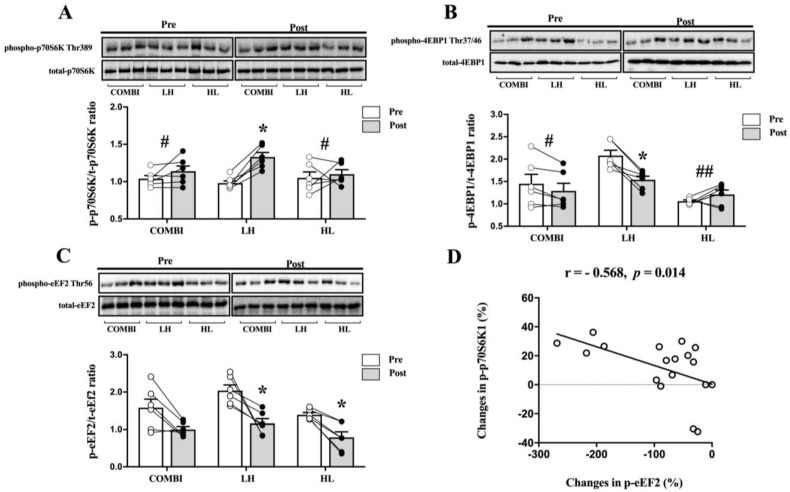
Effect of three RT regimens on phosphorylation of p70S6K1, 4EBP1, and eEF2 in elite weightlifters. (**A**) Representative western blots and quantification of phospho-p70S6K1 to total-p70S6K1 (p-p70S6K1/t-p70S6K1) ratio, (**B**) Phospho-4EBP1 to total-4EBP1 (p-4EBP1/t-4EBP1) ratio, and (**C**) phospho-eEF2 to total-eEF2 (p-eEF2/t-eEF2) ratio. (**D**) Correlation between percentage change in p-p70S6K1/t-p70S6K1 ratio and p-eEF2/t-eEF2 ratio. Each circle, with white and black colors, represents individual data points before and after RT, respectively. Bars represent mean ± SD (n = 6 per group). * *p* < 0.05, from Pre to Post. # *p* < 0.05 and ## *p* < 0.01, compared with LH group.

We found significant differences in the p-p70S6K1/t-p70S6K1 ratio between groups (*p* = 0.027, Figure 3A). The p-p70S6K1/t-p70S6K1 ratio was significantly higher in the LH group compared to the COMBI and HL groups (*p* = 0.043, *p* = 0.021, respectively). 

In addition, only the p-p70S6K1/t-p70S6K1 ratio in the LH group was significantly increased post-RT compared with pre-RT (*p* = 0.028). Significant differences in the p-4EBP1/t-4EBP1 ratio between groups were also revealed (*p* = 0.006, Figure 3B). The p-4EBP-1/t-4EBP-1 ratio was significantly lower in the LH group than in the COMBI and HL groups (*p* = 0.038, *p* = 0.001, respectively), and was significantly decreased only in the LH group post-RT compared with pre-RT (*p* = 0.028). Furthermore, no significant differences in the p-eEF2/t-eEF2 ratio were observed between groups, although it was significantly reduced post-RT compared with pre-RT in the LH and HL groups (*p* = 0.027, *p* = 0.028, respectively, Figure 3C). The p-p70S6K1/t-p70S6K1 ratio was negatively correlated with the p-eEF2/t-eEF2 ratio (r = −0.568, *p* = 0.014, Figure 3D).

### 2.5. Muscle Damage Markers

**Figure 4:**
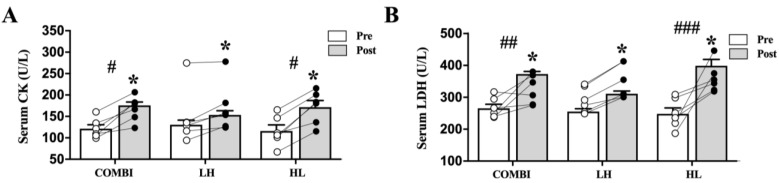
Effect of three RT regimens on muscle damage markers in elite weightlifters. (**A**) The level of CK and (**B**) LDH following 8 weeks of three different RT regimens. Each circle, with white and black colors, represents individual data points before and after RT, respectively. Bars represent mean ± SD (n = 6 per group). * *p* < 0.05 from Pre to Post. # *p* < 0.05, ## *p* < 0.01, and ### *p* < 0.001 compared with LH group.

Significant differences in serum CK and LDH activities between groups (*p* = 0.015 and *p* = 0.004, respectively, Figure 4) were observed. CK activities were significantly lower in the LH group compared to the COMBI and HL groups (*p* = 0.013, *p* = 0.011, respectively). The LDH activities were significantly lower in the LH group compared to the COMBI and HL groups (*p* = 0.000, *p* = 0.006, respectively). In addition, CK and LDH activities were significantly increased post-RT compared with pre-RT in the COMBI, LH, and HL groups (CK: *p* = 0.028, *p* = 0.028, *p* = 0.028, respectively and LDH: *p* = 0.028, *p* = 0.028, *p* = 0.028, respectively).

## 3. Discussion

Elite weightlifters are traditionally encouraged to perform the HL-RT regimen as an effective method to increase muscle mass and strength which potentially leads to augmented muscle damage and diminished performance. Interestingly, several previous studies have also shown similar muscle mass and strength adaptations following HL- and LH-RT [4,5,9]. Nonetheless, as previously mentioned, these studies investigated LH-RT on untrained subjects and/or with an acute intervention. To overcome these limitations, we measured intramuscular anabolic mechanisms and MMS in elite weightlifters after 8 weeks of RT, which allows for a sufficient assessment of the acute effects of different resistance training (RT) loads and repetitions and may better represent the effects of LH-RT on muscle adaptation and thus provide information on whether this exercise modality can be applied to athletes. Here, we found that SMM was increased significantly, accompanied by increased 1-RM in squat and BMS after RT in all groups. This result is consistent with previous studies showing that high-volume RT could also increase muscle mass and strength [4,6,8,16], however, also reporting superior MMS adaptations following HL-RT compared to LH-RT. This discrepancy may be due to the participants’ training status. For example, the subjects of previous studies are entry-level to novice practitioners, who did not demonstrate adaptions in a 1-RM. A previous study suggested that 1-RM testing familiarization is very important when comparing MMS between high-load vs. low-load RT protocols [13]. Therefore, a possible explanation is that the previous subjects who performed HL-RT were likely to have maximal muscle strength greater than that after LH-RT. By contrast, our subjects were already very well familiarized to 1-RM testing (years of experience), thus excluding the confounding influence of a learning effect.

It is well known that increased muscle mass is associated with improvements in muscle strength [17]. Our data revealed that SMM was increased roughly ~3% from baseline in all RT conditions, indicating that a small change in SMM may be sufficient to improve maximal muscle strength. A previous study suggested that minimal changes in exercise performance-related factors, even a 3–5% increase, could have a positive effect on exercise performance in elite athletes, ultimately supporting our hypothesis [18].

Anabolic hormones also play a pivotal role in the regulation of muscle growth. Indeed, RT leads to increases in anabolic hormone levels, which would augment muscle protein synthesis, and ultimately promote muscle hypertrophy [19]. However, other studies have suggested that RT-mediated muscle hypertrophy is not affected by changes in hormone levels [20,21]. This discrepancy may be due to the heterogeneity between types of RT, duration of training, and individual characteristics of the subjects enrolled in these studies. Nevertheless, it is well known that the increase in anabolic hormones in response to RT positively affects muscle function [22,23]. In the current investigation, only GH level was significantly increased across all groups, which confirm the findings of a study using trained men which demonstrated a similar effect of acute high-volume or high-intensity RT [24]. Unlike previous studies, however, the current investigation measured the hormone level at a single point in time, 12 h post-RT, to investigate hormone adaptations following the long-term RT. Thus, our results indicated that 8 weeks of RT may induce hormone adaptations, which are associated with muscle growth, resulting in increased SMM and muscle strength in elite weightlifters. Considering that changes in resting hormone levels following RT may not be detectable for up to 24 weeks [21,25], RT-mediated alterations in circulating hormones remain a controversial measurement. Thus, further research is required to verify the detailed mechanisms by which HL- and LH-RT may influence anabolic hormone concentrations.

The mTOR signaling pathway is a well-known key component of the intramuscular anabolic pathway and has been shown to increase muscle protein synthesis and muscle hypertrophy [26]. Previous studies have suggested that RT stimulates phosphorylation of AKT and/or mTOR, which contributes to the regulation of translation initiation, resulting in increased muscle hypertrophy [9]. The few studies that have compared HL- and LH-RT with respect to mTOR signaling-related proteins presented confounding results [5,24,27,28]. Although it remains unclear why the responses to RT were different in each study, the discrepancies may be due to methodological differences, such as the type of RT, duration (acute vs. long-term), and the timing of protein measurement. Of note, acute RT-induced mTOR signaling has been shown to be highly activated after 24 h or more [28,29]. However, the pathway appears to be more affected by long-term training [26,30]. Therefore, RT-induced muscle hypertrophy, especially long-term and measured 24+ h later, may be critical to determine the net anabolic mechanisms. Nonetheless, these studies focused on changes in AKT and mTOR phosphorylation within a 24 h period and/or in response to acute RT. To overcome these limitations, we analyzed mTOR signaling proteins following 8 weeks of RT, 48 h after the end of the regimens. Our findings indicate that only the p-AKT/t-AKT ratio was significantly lower in the HL group compared to that in other training groups and decreased significantly post-RT compared to pre-RT. Moreover, the p-mTOR/t-mTOR ratio did not change following RT between groups or with time. Although it is unknown whether these protein levels were reduced or unchanged following RT, these results may be associated with the training status of our subjects. Previous studies have suggested that the activation of mTOR-related proteins is reduced or unchanged following RT in well-trained subjects compared with untrained subjects [24,29]. The participants in our study were elite weightlifting athletes who regularly performed RT 3–4 h daily, which may account for the unaffected AKT/mTOR protein levels following RT. Interestingly, a previous study suggested that without the AKT/mTOR-dependent pathway, activation of p70S6K1 and 4E-BP1, known to be downstream of mTOR, can also be influenced by RT. Thus, it can be assumed that the RT-induced increase in SMM may be mediated by an AKT/mTOR-independent pathway in well-trained athletes. Surprisingly, we found that changes in the p-p70S6K1/t-p70S6K1 ratio were greater in the LH group compared to the other training groups and was only elevated post-RT in the LH group. This result is consistent with previous studies showing that high-repetition RT increased phosphorylation of p70S6K1 compared with low-repetition RT, despite no adjustment in AKT phosphorylation [27,31]. Although it is not clear how LH-RT increased p70S6K1 phosphorylation, it is possible that the number of RT repetitions was a critical underlying factor. A previous study suggested that the RT-induced increase in p70S6K1 phosphorylation in animal and human muscles depended on RT repetition, especially at high volumes [31]. Thus, our data support the notion that p70S6K1 phosphorylation may not be affected by RT-mediated AKT/mTOR phosphorylation but rather high-repetition RT in well-trained athletes [32].

4E-BP1, another molecule downstream of mTOR that controls translation initiation, was also upregulated through RT [9]. However, the effect of high-repetition RT on phosphorylation of 4E-BP1 is controversial, with a few studies in animals and humans showing no change [4,31] or increased levels [10]. In contrast to previous studies, the current investigation found that the p-4E-BP1/t-4E-BP1 ratio was lower following RT in the LH group compared with the other training groups and was reduced significantly following RT in the LH group only. Although this difference was puzzling, previous studies have suggested various responses of 4E-BP1 phosphorylation following muscle activity [32]. Thus, a future detailed study is needed to verify the differential regulation of 4E-BP1 phosphorylation in the skeletal muscle of well-trained athletes following LH-RT.

Notably, eEF2, a downstream factor in the mTOR signaling pathway, regulates protein translation. Generally, phosphorylation of eEF2 results in an inactivated isoform, impairing the binding of eEF2 to ribosomes and resulting in inhibited protein translation [33]. A previous study suggested that eEF2 phosphorylation (inactivated form) was reduced following acute RT, which contributed to the maintenance of muscle protein synthesis and hypertrophy [34]. However, other studies did not identify any change in eEF2 phosphorylation following RT [35]. To our knowledge, there are only two studies that have shown a reduced or unchanged eEF2 phosphorylation following high-repetition RT compared with low-repetition RT [10,27]. Again, these discrepancies may be due to differences in RT protocols (e.g., duration, number of repetitions, and intensity), participant training experience, and/or the timing of protein measurement. In accordance with the findings of the two latter studies, we found that the p-eEF2/t-eEF2 ratio was significantly reduced following RT in the LH and HL groups. This decreased resting activation of eEF2 could explain the higher resting protein translation observed after RT in well-trained individuals. In particular, a previous study suggested that p70S6K1 activation can reduce eEF2 phosphorylation, which may increase translation elongation [36]. Thus, our results implied that decreased eEF2 phosphorylation by LH-RT may be associated with increased p70S6k1 phosphorylation, as verified by the negative correlation between p70S6k1 and eEF2. Taken together, our findings demonstrated that LH-RT could decrease eEF2 phosphorylation, which may lead to subsequent protein translation, thereby increasing SMM and muscle strength in elite weightlifters.

Excessive HL-RT over a long period of time is likely to cause muscle damage, which may decrease exercise performance [3,37]. Therefore, elite weightlifters and their coaches endeavor to restore muscle damage after excessive HL-RT. Interestingly, although CK and LDH activities increased after all RT interventions compared with pre-RT, they were significantly lower in the LH group compared to the other RT groups, confirming previous observations [24]. This finding suggested that compared with HL-RT, LH-RT may also reduce RT-induced muscle damage, which may contribute to reduced recovery time and fatigue and ultimately improve exercise performance following RT.

Although we suggest a relationship between the three RT regimens and muscle hypertrophy-related factors, the effects of RT on the underlying mechanisms of muscle physiology remain unclear. Previous studies have shown that phosphorylation of p70S6K1 was not changed following LH-RT but increased following HL-RT [5,24]. Moreover, RT also induces protein synthesis and muscle hypertrophy through extracellular signal-regulated kinase (ERK) and its downstream p90 ribosomal S6 kinase (p90RSK), bypassing the mTOR signaling pathway [4,12]. Future research is required to determine the precise molecular mechanisms by which LH-RT induces activation of p70S6K1 and p90RSK in well-trained human skeletal muscle. In addition, we did not thoroughly explore COMBI-RT, which did not result in mTOR signaling-related differences. However, this type of RT increased SMM, GH, and muscle strength. Furthermore, COMBI-RT, such as interval training, can also exert a beneficial effect on cardiovascular adaptation and fatigue recovery [38]. Since these factors are also critical for weightlifters, further studies are required to clarify these mechanisms.

The main limitation of our study is the small sample size, which was insufficient to verify that increased maximal muscle strength was due to LH-RT. The small sample size is due to the overall small population of world-class weightlifters, and a general hesitation of most weightlifters to change their RT program in case of injury or loss of exercise performance. However, given the expertise and distinct nature of the unique cohort, the limited sample size in three different groups contributes significant value to the study, and their participation provides insights with a broad spectrum of training modalities that may not be readily obtainable from a larger, less-specialized cohort. Thus, further studies in a larger elite weightlifter cohort may be required to confirm these results. Another potential limitation of our study is that we measured SMM using BIA, which is not considered a direct measurement compared with standard magnetic resonance imaging (MRI). However, MRI is expensive, time-consuming, and must be performed by skilled technicians in a medical setting. Conversely, the BIA method is quick (i.e., within 2 min), highly reproducible (coefficient of variation, <5%), fairly inexpensive, and can be performed without the need of skilled professionals. Moreover, Wang et al. [39] have suggested that BIA provides accurate and reliable estimations of SMM compared with standard MRI methods, further supporting the applicability of BIA in assessing muscle mass in athletes, as underscored by Bauer et al.’s systematic review [40]. Thus, we believe that our SMM data obtained by BIA are reliable evidence of RT-induced hypertrophy.

Taken together, our findings revealed that 8 weeks of LH-RT increases skeletal muscle mass and MMS, similarly to HL-RT, while reducing RT-induced muscle damage markers in elite weightlifters. These results may provide elite weightlifters and coaches effective alternative methodologies in the development of RT programs to improve exercise performance.

## 4. Materials and Methods

### 4.1. Subjects

**Figure 5:**
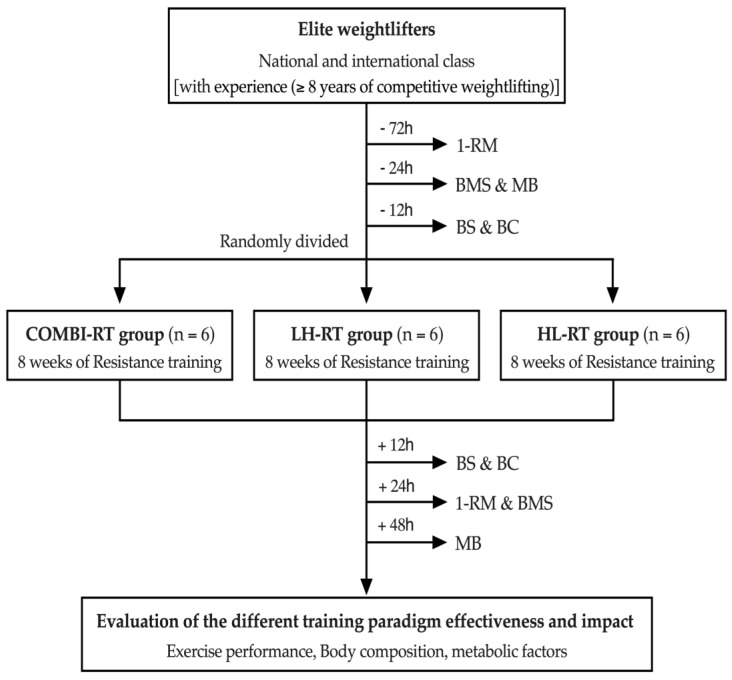
Experimental design. Experimental timeline illustrating the assessment of three different RT regimens. One-repetition maximum (1-RM), muscle biopsy (MB), back muscle strength (BMS), blood sampling (BS), and body composition (BC).

Eighteen male weightlifters, members of the Korean National Sport University Team (national and international class; age: 20.2 ± 0.2 years; body weight: 86.4 ± 4.0 kg; height: 1.7 ± 0.1 m; snatch record: 123.3 ± 14.7; squat record: 212.2 ± 27.2; clean and jerk: 168.6 ± 22.4), volunteered to participate in the study. Athletes were enrolled based on experience (≥8 years of competitive weightlifting) and accolades (those who have won competitions in their respective weight class). The subjects were randomly divided into three groups using a randomized block design: (a) COMBI group; (b) LH group; (c) HL group (Table 2, Figure 5).

### 4.2. Resistance Training Protocols

The subjects were familiarized with all testing procedures prior to the study and participated in a supervised RT program throughout the study. RT programs that were followed for each intervention group (3–4 h/day, 3 days/week [Monday, Wednesday, and Friday], 8 weeks). Briefly, for each training method (LH-RT and HL-RT), two programs (displayed as A and B) were completed in alternating workout sessions, which consisted of four different lifting exercises. Exercises in program A consisted of lifts focused on the movements involved in a snatch such as front and back squat press, snatch, snatch pull, and power snatch, while the exercises in program B consisted of lifts focused on the movements involved in a clean and jerk such as front and back push press, clean and jerk, clean pull, and power clean and jerk. Weightlifters performed all of the prescribed repetitions and sets before progressing to the next intensity (LH-RT: 30, 40, 50, and 60% of 1-RM; HL-RT: 60, 70, 80, and 90% of 1-RM), and all intensities were completed before progressing to the next exercise. The COMBI group followed the same prescription as both the LH-RT and HL-RT groups; however, the intensity and volume paradigms of the training sessions alternated between those of each training group (i.e., LH-RT program A, LH-RT program B, HL-RT program A, and HL-RT program B, repeated for the training period). The rest time for each LH-RT was 30–90 s, and that for each HL-RT was 60–180 s.

### 4.3. Determination of 1-RM and BMS

All participants performed 1-RM tests for the back squat and snatch exercises. Before and after the 8-week training period, each participant performed a warm-up set at approximately 40–60% of his perceived maximum, followed by 60–70% (2 repetitions), 70–80% (2 repetitions), 90% (1 repetition), 95–100% (1 repetition), and 105% (1 repetition) to determine the 1-RM with 30–120 s of rest between each lift. To investigate the test-retest reproducibility, we determined the Pearson correlation coefficient (squat: r = 0.991, *p* < 0.001, snatch: r = 0.973, *p* < 0.001). BMS, maximal isometric strength of the trunk muscles such as the external oblique abdominal, internal oblique abdominal, transverse abdominal, erector spinae, and latissimus dorsi muscle, which can increase intra-abdominal strength, leading to improve exercise performance, was measured using a digital back muscle strength instrument (TKK 5402, Takei Scientific Instruments Co., Nigata, Japan). All participants performed a standardized warm-up, and then they stood on a plate with 30° lumbar flexion and grasped the handle with straight knees and back. Upon commencement, participants pulled the handle backward, engaging the back muscles as much as they could. This test was performed twice, and the average force from the two trials was recorded.

### 4.4. Body Composition

Body weight (BW, kg), skeletal muscle mass (SMM, kg), and body fat percentage (BF%) were measured using eight-polar bioelectrical impedance analysis (BIA) with multiple impedance frequencies (Inbody 720, Biospace Co., Seoul, Republic of Korea). In brief, all subjects were instructed to limit food and water intake for at least 8 h and empty their bladder 4 h before the measurement. The device was calibrated according to the manufacturer’s instructions, and subjects wore light clothing and removed all metal items to ensure accurate body composition measurement. Afterward, height was measured using a fatness measuring system (Dong-Sahn Jenix, Seoul, Republic of Korea). During measurement, each subject stood with his soles in contact with the foot electrodes and gripped the hand electrodes. The arms were fully extended and abducted approximately 20–30° laterally. After the measurement, all data were electronically imported into Excel using InBody software (BioSpace, West Des Moines, IA, USA).

### 4.5. Muscle Biopsy

Muscle samples were obtained from the middle portion of the vastus lateralis muscle by the percutaneous needle biopsy technique 16. The pre-RT biopsies were collected from the left leg, with no physical activity for 72 h prior to the biopsy. The post-training (after 8 weeks) biopsies were obtained from the same left leg 48 h after the final training session. Muscle samples were removed, cleaned of blood, and frozen within 1 min in liquid nitrogen (−80 °C) for subsequent protein analysis.

### 4.6. Blood Collection and Biochemical Analyses

Blood samples were drawn from the antecubital vein via multiple venipunctures at 12 h before and 12 h after the 8-week intervention period for the determination of insulin, insulin-like growth factor 1 (IGF-1), growth hormone (GH), CK, and LDH. All samples were sent to Green Cross Co. (Yongin, Republic of Korea) for the determination of basal hormone concentrations using an ELISA kit for serum insulin (Count-A-Count; DPC, Los Angeles, CA, USA), plasma IGF- I (Quantikine; R&D Systems, Inc., Minneapolis, MN, USA), and serum GH (Pharmacia Diagnostics, Uppsala, Sweden). In addition, serum CK and LDH activity, which are key markers of muscle damage, were analyzed by a commercial kit (Roche, Germany) using an automated analyzer, Cobas 8000 (Roche c702, Mannheim, Germany). All samples were assayed according to the manufacturer’s instructions, and the results represent values obtained from duplicate experiments.

### 4.7. Western Blotting

Proteins (20–30 μg) were separated by 10–12% SDS-polyacrylamide gel electrophoresis for 100–150 min and transferred to polyvinylidene fluoride (PVDF) membranes (Millipore, Boston, MA, USA) for 1–2 h at 90–120 V. These membranes were blocked in a 3–5% (*w*/*v*) BSA solution for 60–90 min at room temperature and incubated overnight at 4 °C with primary antibodies: total-AKT, phospho-AKT, total-mTOR, phospho-mTOR, total-4EBP1, phospho-4EBP1, total-p70S6K1, phospho-p70S6K1, total-eEf2, and phospho-eEf2 from Cell Signaling Technologies (Danvers, MA, USA; 1:1000). The membranes were washed five times for 10 min with TBS with 0.1% Tween-20 (TBS-T) and incubated at room temperature with secondary antibodies: goat anti-rabbit (Invitrogen, Carlsbad, CA, USA; 1:5000), rabbit anti-goat (Life Technologies, Carlsbad, CA, USA; 1:5000), and goat anti-mouse (Santa Cruz Biotechnology; 1:5000). The membranes were then washed five times again for 10 min with TBS-T. Immunoreactive proteins were detected with enhanced chemiluminescence reagents (Santa Cruz, Biotechnology, CA, USA) following the manufacturer’s instructions. Protein band densities were determined using the ChemiDoc XRS system (Bio-Rad, Hercules, CA, USA).

### 4.8. Statistical Analysis

Statistical analyses were carried out with SPSS (version 22.0, SPSS Inc., Chicago, IL, USA), and data are presented as means ± SD. The Shapiro–Wilk test was performed to confirm whether the variables were normally distributed, and Levene’s test was used to analyze the homogeneity of variance. Because of random violations of the normal distribution assumption, nonparametric tests were conducted. Differences between the groups in RT-induced change (pre-post) were analyzed with the Kruskal–Wallis test, followed by the rank-based version of Tukey’s Honestly Significant Difference (HSD). Changes between groups were assessed by the Wilcoxon matched-pairs signed-rank test. To determine correlations, Spearman’s rank correlation coefficient test was used. Differences were considered statistically significant if *p* < 0.05.

## 5. Conclusions

This study challenges the conventional preference for the HL-RT regimen among elite weightlifters. The LH-RT demonstrated comparable muscle mass and strength adaptations to HL-RT, providing a noteworthy alternative to the traditional approach. Elite weightlifters undergoing LH-RT exhibited increased SMM, enhanced 1-RM, and a significant rise in p70S6K1 phosphorylation, indicating an AKT/mTOR-independent pathway. Moreover, LH-RT showed the reduced muscle damage markers, suggesting potential benefits in minimizing recovery time for elite weightlifters. While acknowledging limitations such as a small sample size and the use of BIA, our findings suggest LH-RT as a promising strategy for elite weightlifters. Future research with larger cohorts and comparison to MRI is warranted to validate these insights.

In summary, our study supports the efficacy of LH-RT, challenging the established paradigm and offering a valuable alternative for optimizing training programs and improving exercise performance in elite weightlifters.

## Figures and Tables

**Table 1:** Body composition and anabolic hormones of the three differences RE training in elite weightlifters.

	COMBI (n = 6)		LH (n = 6)		HL (n = 6)	
	Pre	Post	Pre	Post	Pre	Post
BW (kg)	81.4 ± 7.4	82.8 ± 7.3	92.0 ± 7.2	93.0 ± 7.8	85.9 ± 6.7	87.6 ± 7.2
BF (%)	18.0 ± 1.7	17.9 ± 1.8	24.0 ± 2.5	23.0 ± 2.6	19.2 ± 1.7	18.6 ± 1.8
SMM (kg)	38.2 ± 3.8	39.1 ± 3.6 *	40.4 ± 2.4	41.7 ± 2.4 *	38.9 ± 3.2	39.9 ± 3.4 *
Insulin(μU/mL)	10.0 ± 2.7	11.6 ± 3.6	12.7 ± 1.9	13.1 ± 3.3	8.7 ± 1.0	7.2 ± 0.8
IGF-1 (ng/mL)	231.3 ± 28.7	272.5 ± 44.6	203.7 ± 11.5	215.7 ± 8.2	203.8 ± 19.0	216.3 ± 28.6
GH (ng/mL)	0.7 ± 0.1	1.2 ± 0.2 *	0.7 ± 0.1	1.1 ± 0.1 *	0.5 ± 0.1	0.9 ± 0.2 *

Values are means ± SD (n = 6 per group). Main time effect: * *p* < 0.05, pre- versus post-RT period in the within groups. BW: body weight, BF: body fat, SMM: skeletal muscle mass, IGF-1: insulin growth factor-1, GH: growth hormone.

**Table 2:** Subject characteristics.

	COMBI (n = 6)	LH (n = 6)	HL (n = 6)	*p*-Value
Age (years)	20.0 ± 1.3	20.8 ± 0.8	19.8 ± 0.8	0.161
Experience (years)	9.83 ± 1.0	9.8 ± 1.2	8.8 ± 1.2	0.277
Height (cm)	171.3 ± 8.2	174.5 ± 7.6	171.0 ± 9.4	0.801
BW (kg)	81.4 ± 18.2	92.0 ± 17.6	85.9 ± 16.4	0.587
BF (%)	18.0 ± 4.3	24.0 ± 6.2	19.2 ± 4.3	0.142
SMM (kg)	38.2 ± 9.3	40.4 ± 5.8	38.9 ± 8.0	0.970
1-RM of back squat (kg)	206.0 ± 35.6	219.0 ± 29.7	211.7 ± 16.0	0.972
1-RM of snatch (kg)	116.7 ± 20.6	127.7 ± 8.9	125.5 ± 12.2	0.430
BMS (kg)	172.8 ± 33.9	181.9 ± 27.4	173.7 ± 9.3	0.619

BW: body weight, BF: body fat, SMM: skeletal muscle mass, 1-RM: one-repetition maximum, BMS: back muscle strength. Values are means ± SD (n = 6 per group).

## Data Availability

The dataset supporting the conclusions of this article is available from the corresponding author on reasonable request.

## References

[1] Garhammer J., Takano B. (1992). Training for Weightlifting. Strength Power Sport..

[2] Calhoon G., Fry A.C. (1999). Injury Rates and Profiles of Elite Competitive Weightlifters. J. Athl. Train..

[3] Le Meur Y., Hausswirth C., Natta F., Couturier A., Bignet F., Vidal P.P. (2013). A Multidisciplinary Approach to Overreaching Detection in Endurance Trained Athletes. J. Appl. Physiol..

[4] Ogasawara R., Loenneke J.P., Thiebaud R.S., Abe T. (2013). Low-Load Bench Press Training to Fatigue Results in Muscle Hypertrophy Similar to High-Load Bench Press Training. Int. J. Clin. Med..

[5] Schoenfeld B.J., Peterson M.D., Ogborn D., Contreras B., Sonmez G.T. (2015). Effects of Low- vs. High-Load Resistance Training on Muscle Strength and Hypertrophy in Well-Trained Men. J. Strength Cond. Res..

[6] Martinho D.V., Nobari H., Faria A., Field A., Duarte D., Sarmento H. (2022). Oral Branched-Chain Amino Acids Supplementation in Athletes: A Systematic Review. Nutrients.

[7] Burd N.A., Mitchell C.J., Churchward-Venne T.A., Phillips S.M. (2012). Bigger Weights May Not Beget Bigger Muscles: Evidence from Acute Muscle Protein Synthetic Responses After Resistance Exercise. Appl. Physiol. Nutr. Metab..

[8] Campos G.E., Luecke T.J., Wendeln H.K., Toma K., Hagerman F.C., Murray T.F., Ragg K.E., Ratamess N.A., Kraemer W.J., Staron R.S. (2002). Muscular Adaptations in Response to Three Different Resistance-Training Regimens: Specificity of Repetition Maximum Training Zones. Eur. J. Appl. Physiol..

[9] Agergaard J., Bülow J., Jensen J.K., Reitelseder S., Drummond M.J., Schjerling P., Scheike T., Serena A., Holm L. (2017). Light-Load Resistance Exercise Increases Muscle Protein Synthesis and Hypertrophy Signaling in Elderly Men. Am. J. Physiol. Endocrinol. Metab..

[10] Burd N.A., Holwerda A.M., Selby K.C., West D.W., Staples A.W., Cain N.E., Cashaback J.G., Potvin J.R., Baker S.K., Phillips S.M. (2010). Resistance Exercise Volume Affects Myofibrillar Protein Synthesis and Anabolic Signalling Molecule Phosphorylation in Young Men. J. Physiol..

[11] Burd N.A., West D.W., Staples A.W., Atherton P.J., Baker J.M., Moore D.R., Holwerda A.M., Parise G., Rennie M.J., Baker S.K. (2010). Low-Load High Volume Resistance Exercise Stimulates Muscle Protein Synthesis More than High-Load Low Volume Resistance Exercise in Young Men. PLoS ONE.

[12] Moore D.R., Kelly R.P., Devries M.C., Churchward-Venne T.A., Phillips S.M., Parise G., Johnston A.P. (2018). Low-Load Resistance Exercise During Inactivity Is Associated with Greater Fibre Area and Satellite Cell Expression in Older Skeletal Muscle. J. Cachexia Sarcopenia Muscle.

[13] Nóbrega S.R., Ugrinowitsch C., Pintanel L., Barcelos C., Libardi C.A. (2018). Effect of Resistance Training to Muscle Failure vs. Volitional Interruption at High- and Low-Intensities on Muscle Mass and Strength. J. Strength Cond. Res..

[14] Sale D.G. (1988). Neural Adaptation to Resistance Training. Med. Sci. Sports Exerc..

[15] Wernbom M., Augustsson J., Thomeé R. (2007). The Influence of Frequency, Intensity, Volume and Mode of Strength Training on Whole Muscle Cross-Sectional Area in Humans. Sports Med..

[16] Mangine G.T., Hoffman J.R., Gonzalez A.M., Townsend J.R., Wells A.J., Jajtner A.R., Beyer K.S., Boone C.H., Miramonti A.A., Wang R. (2015). The Effect of Training Volume and Intensity on Improvements in Muscular Strength and Size in Resistance-Trained Men. Physiol. Rep..

[17] Maughan R.J., Watson J.S., Weir J. (1983). Relationships Between Muscle Strength and Muscle Cross-Sectional Area in Male Sprinters and Endurance Runners. Eur. J. Appl. Physiol. Occup. Physiol..

[18] Barnett A., Cerin E., Reaburn P., Hooper S. (2010). The Effects of Training on Performance and Performance-Related States in Individual Elite Athletes: A Dynamic Approach. J. Sports Sci..

[19] Gonzalez A.M., Hoffman J.R., Stout J.R., Fukuda D.H., Willoughby D.S. (2016). Intramuscular Anabolic Signaling and Endocrine Response Following Resistance Exercise: Implications for Muscle Hypertrophy. Sports Med..

[20] Schroeder E.T., Villanueva M., West D.D., Phillips S.M. (2013). Are Acute Post-resistance Exercise Increases in Testosterone, Growth Hormone, and IGF-1 Necessary to Stimulate Skeletal Muscle Anabolism and Hypertrophy?. Med. Sci. Sports Exerc..

[21] West D.W., Burd N.A., Tang J.E., Moore D.R., Staples A.W., Holwerda A.M., Baker S.K., Phillips S.M. (2010). Elevations in Ostensibly Anabolic Hormones with Resistance Exercise Enhance Neither Training-Induced Muscle Hypertrophy nor Strength of the Elbow Flexors. J. Appl. Physiol..

[22] Kraemer W.J., Ratamess N.A. (2005). Hormonal Responses and Adaptations to Resistance Exercise and Training. Sports Med..

[23] West D.W., Phillips S.M. (2012). Associations of Exercise-Induced Hormone Profiles and Gains in Strength and Hypertrophy in a Large Cohort After Weight Training. Eur. J. Appl. Physiol..

[24] Gonzalez A.M., Hoffman J.R., Townsend J.R., Jajtner A.R., Wells A.J., Beyer K.S., Willoughby D.S., Oliveira L.P., Fukuda D.H., Fragala M.S. (2015). Association Between Myosin Heavy Chain Protein Isoforms and Intramuscular Anabolic Signaling Following Resistance Exercise in Trained Men. Physiol. Rep..

[25] McCall G.E., Byrnes W.C., Fleck S.J., Dickinson A., Kraemer W.J. (1999). Acute and Chronic Hormonal Responses to Resistance Training Designed to Promote Muscle Hypertrophy. Can. J. Appl. Physiol..

[26] Bodine S.C., Stitt T.N., Gonzalez M., Kline W.O., Stover G.L., Bauerlein R., Zlotchenko E., Scrimgeour A., Lawrence J.C., Glass D.J. (2001). Akt/mTOR Pathway Is a Crucial Regulator of Skeletal Muscle Hypertrophy and Can Prevent Muscle Atrophy In Vivo. Nat. Cell Biol..

[27] Ahtiainen J.P., Walker S., Silvennoinen M., Kyröläinen H., Nindl B.C., Häkkinen K., Nyman K., Selänne H., Hulmi J.J. (2015). Exercise Type and Volume Alter Signaling Pathways Regulating Skeletal Muscle Glucose Uptake and Protein Synthesis. Eur. J. Appl. Physiol..

[28] Ogasawara R., Sato K., Matsutani K., Nakazato K., Fujita S. (2014). The Order of Concurrent Endurance and Resistance Exercise Modifies MTOR Signaling and Protein Synthesis in Rat Skeletal Muscle. Am. J. Physiol. Endocrinol. Metab..

[29] Wilkinson S.B., Phillips S.M., Atherton P.J., Patel R., Yarasheski K.E., Tarnopolsky M.A., Rennie M.J. (2008). Differential Effects of Resistance and Endurance Exercise in the Fed State on Signalling Molecule Phosphorylation and Protein Synthesis in Human Muscle. J. Physiol..

[30] Goodman C.A., Frey J.W., Mabrey D.M., Jacobs B.L., Lincoln H.C., You J.S., Hornberger T.A. (2011). The Role of Skeletal Muscle mTOR in the Regulation of Mechanical Load-Induced Growth. J. Physiol..

[31] Ogasawara R., Arihara Y., Takegaki J., Nakazato K., Ishii N. (2017). Relationship Between Exercise Volume and Muscle Protein Synthesis in a Rat Model of Resistance Exercise. J. Appl. Physiol..

[32] Liu Y., Vertommen D., Rider M.H., Lai Y.C. (2013). Mammalian Target of Rapamycin-Independent S6K1 and 4E-BP1 Phosphorylation during Contraction in Rat Skeletal Muscle. Cell. Signal..

[33] Susorov D., Zakharov N., Shuvalova E., Ivanov A., Egorova T., Shuvalov A., Shatsky I.N., Alkalaeva E. (2018). Eukaryotic Translation Elongation Factor 2 (eEF2) Catalyzes Reverse Translocation of the Eukaryotic Ribosome. J. Biol. Chem..

[34] West D.W., Baehr L.M., Marcotte G.R., Chason C.M., Tolento L., Gomes A.V., Bodine S.C., Baar K. (2016). Acute Resistance Exercise Activates Rapamycin-Sensitive and -Insensitive Mechanisms That Control Translational Activity and Capacity in Skeletal Muscle. J. Physiol..

[35] Fernandez-Gonzalo R., Lundberg T.R., Tesch P.A. (2013). Acute Molecular Responses in Untrained and Trained Muscle Subjected to Aerobic and Resistance Exercise Training Versus Resistance Training Alone. Acta Physiol..

[36] Wang X., Proud C.G. (2006). The mTOR Pathway in the Control of Protein Synthesis. Physiology.

[37] Meeusen R., Duclos M., Foster C., Fry A., Gleeson M., Nieman D., Raglin J., Rietjens G., Steinacker J., Urhausen A. (2013). Prevention, Diagnosis, and Treatment of the Overtraining Syndrome: Joint Consensus Statement of the European College of Sport Science and the American College of Sports Medicine. Med. Sci. Sports Exerc..

[38] Stavrinou P.S., Bogdanis G.C., Giannaki C.D., Terzis G., Hadjicharalambous M. (2018). High-Intensity Interval Training Frequency: Cardiometabolic Effects and Quality of Life. Int. J. Sports Med..

[39] Wang J.G., Zhang Y., Chen H.E., Li Y., Cheng X.G., Xu L., Guo Z., Zhao X.S., Sato T., Cao Q.Y. (2013). Comparison of Two Bioelectrical Impedance Analysis Devices with Dual Energy X-ray Absorptiometry and Magnetic Resonance Imaging in the Estimation of Body Composition. J. Strength Cond. Res..

[40] Bauer P., Majisik A., Mitter B., Csapo R., Tschan H., Hume P., Martínez-Rodríguez A., Makivic B. (2023). Body Composition of Competitive Bodybuilders: A Systematic Review of Published Data and Recommendations for Future Work. J. Strength Cond. Res..

